# Supplementary material for: Cytotoxic, Antimicrobial, Antioxidant Properties and Effects on Cell Migration of Phenolic Compounds of Selected Transylvanian Medicinal Plants
Source: Antioxidants (Basel). 2020 Feb 18;9(2):166. doi: 10.3390/antiox9020166 (PMC7070992; doi:10.3390/antiox9020166)
Supplement: Supplementary file 1 [file antioxidants-09-00166-s001.zip › Supplementary Material proof.docx]

Cytotoxic, Antimicrobial, Antioxidant Properties and Effects on Cell Migration of Phenolic Compounds of Selected Transylvanian Medicinal Plants

Supplementary Material

Rita Csepregi^1,2^, Viktória Temesfői^1,2^, Sourav Das^1,2^, Ágnes Alberti^3^, Csenge Anna Tóth^3^, Róbert Herczeg^2^, Nóra Papp^4^ and Tamás Kőszegi^1,2*^

^1^ Department of Laboratory Medicine, University of Pécs, Medical School, Ifjúság u. 13, 7624 Pécs, Hungary; [ritacsepregi93@gmail.com](mailto:ritacsepregi93@gmail.com) (R.C.); [vtemesfoi@gmail.com](mailto:vtemesfoi@gmail.com) (V.T.); [pharma.souravdas@gmail.com](mailto:pharma.souravdas@gmail.com) (S.D); [tamas.koszegi@aok.pte.hu](mailto:tamas.koszegi@aok.pte.hu) (T.K)

^2^ János Szentágothai Research Center, University of Pécs, Ifjúság u. 20, 7624 Pécs, Hungary; [herczeg.robert@pte.hu](mailto:herczeg.robert@pte.hu) (R.H.)

^3^ Department of Pharmacognosy, Semmelweis University, Üllői út 26, 1085 Budapest, Hungary; [albertiagnes@gmail.com](mailto:albertiagnes@gmail.com) (A.A); [csenge512@gmail.com](mailto:csenge512@gmail.com) (C.A.T)

^4^ Department of Pharmacognosy, University of Pécs, Faculty of Pharmacy, Rókus u. 2, 7624 Pécs, Hungary; [nora4595@gamma.ttk.pte.hu](mailto:nora4595@gamma.ttk.pte.hu) (N.P.)

***** Correspondence: [tamas.koszegi@aok.pte.hu](mailto:tamas.koszegi@aok.pte.hu); Tel.: +36-30-491-7719; Fax: +36-72-536-121


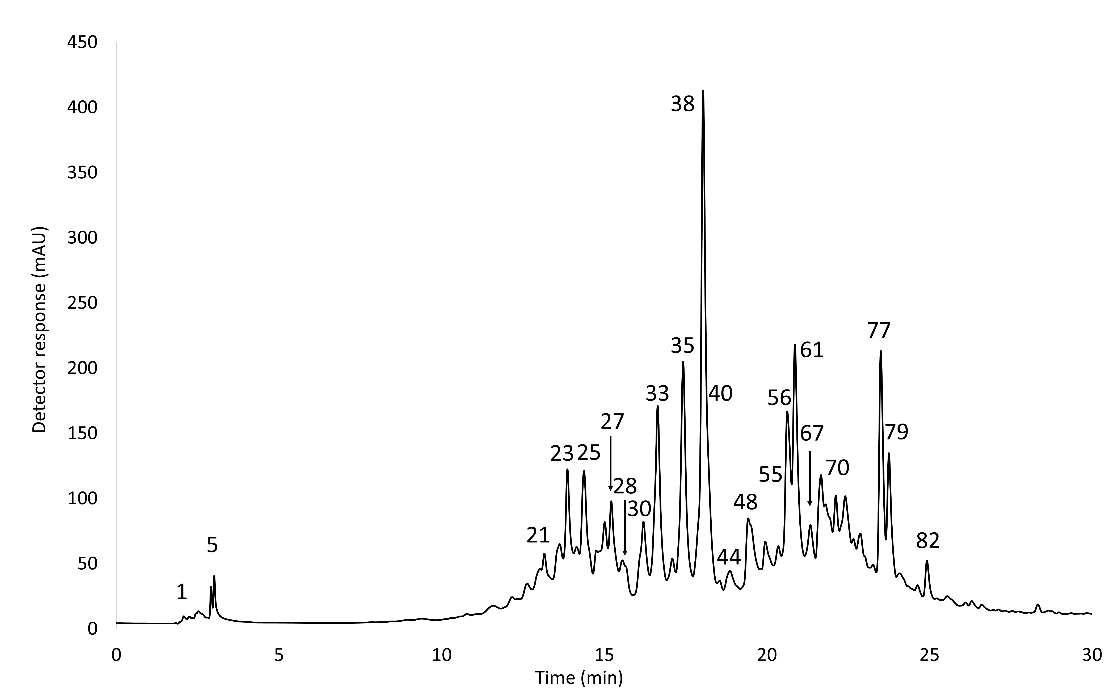


**Figure S1**: HPLC-DAD chromatogram of *A. vulneraria* 50% (v/v) ethanolic extract, detection wavelength: 280 nm. Numbering of peaks refers to data shown in Table 1.


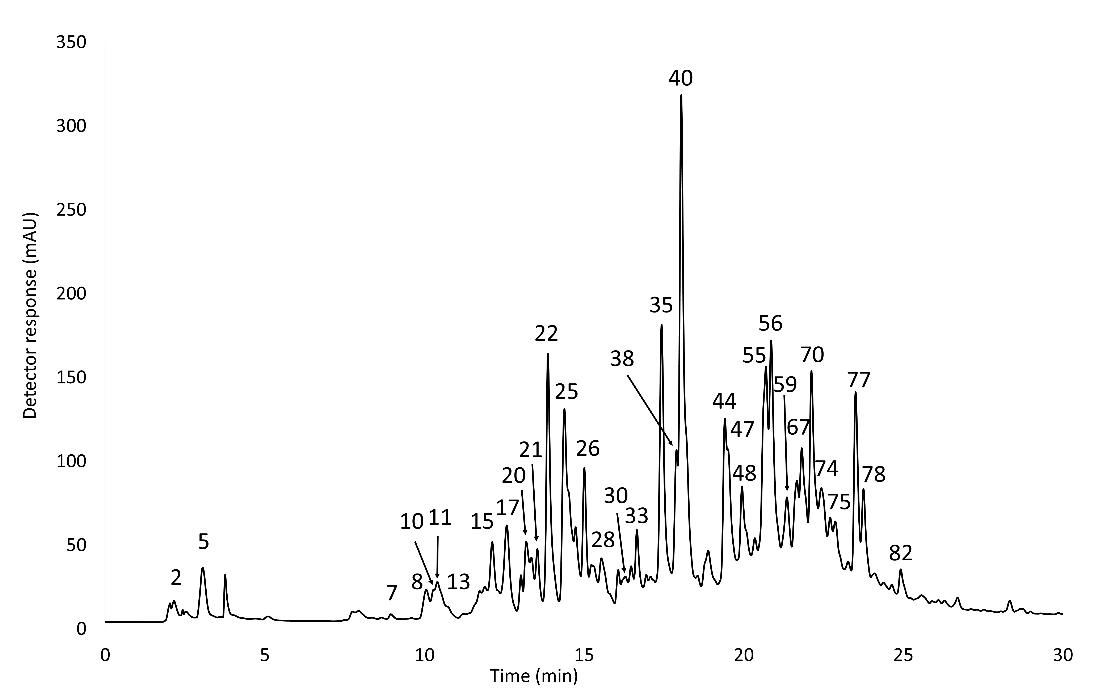


**Figure S2**: HPLC-DAD chromatogram of *A. vulneraria* aqueous extract, detection wavelength: 280 nm. Numbering of peaks refers to data shown in Table 1.


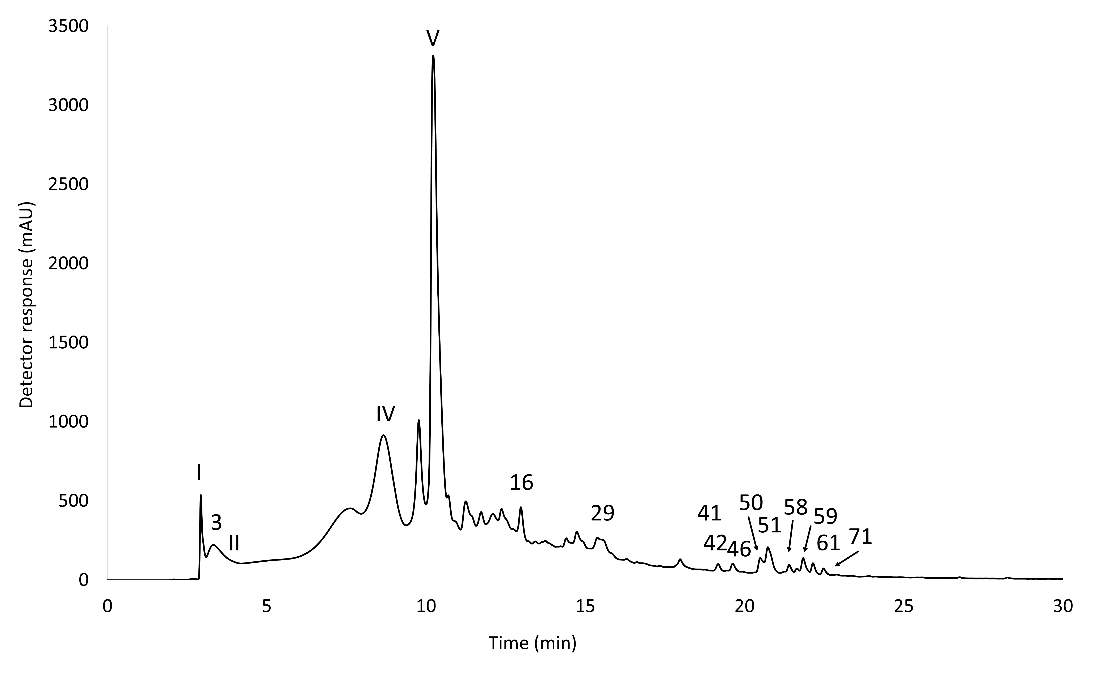


**Figure S3**: HPLC-DAD chromatogram of *F. magellanica* 50% (v/v) ethanolic extract, detection wavelength: 280 nm. Numbering of peaks refers to data shown in Table 1 and 2.


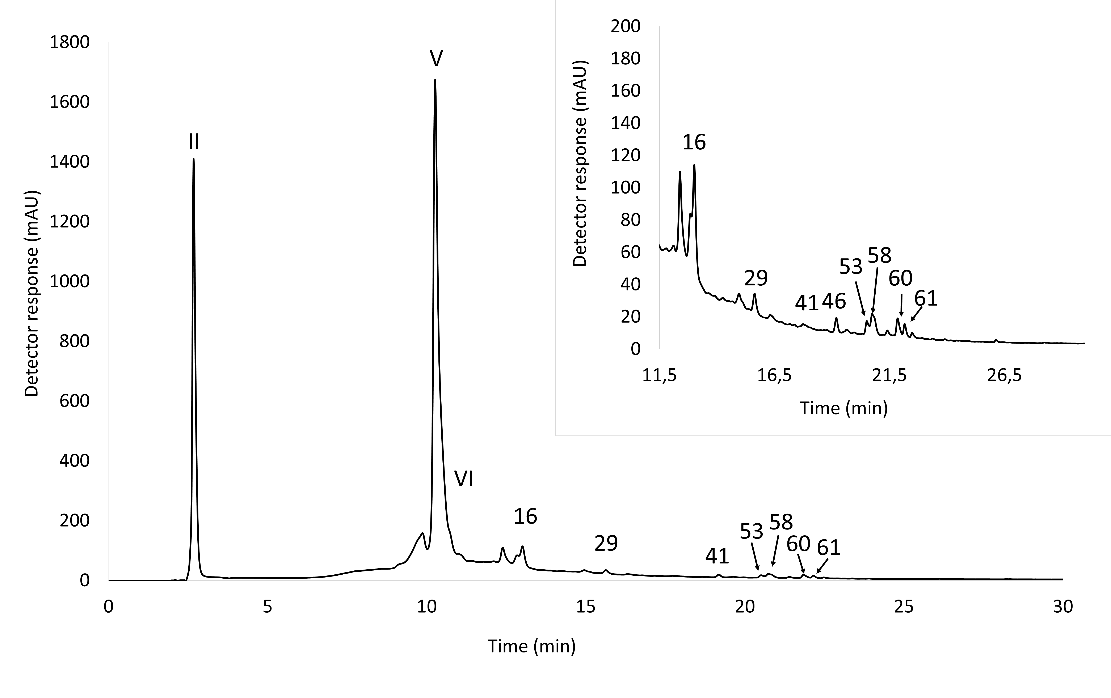


**Figure S4**: HPLC-DAD chromatogram of *F. magellanica* aqueous extract, detection wavelength: 280 nm. Numbering of peaks refers to data shown in Table 1 and 2.


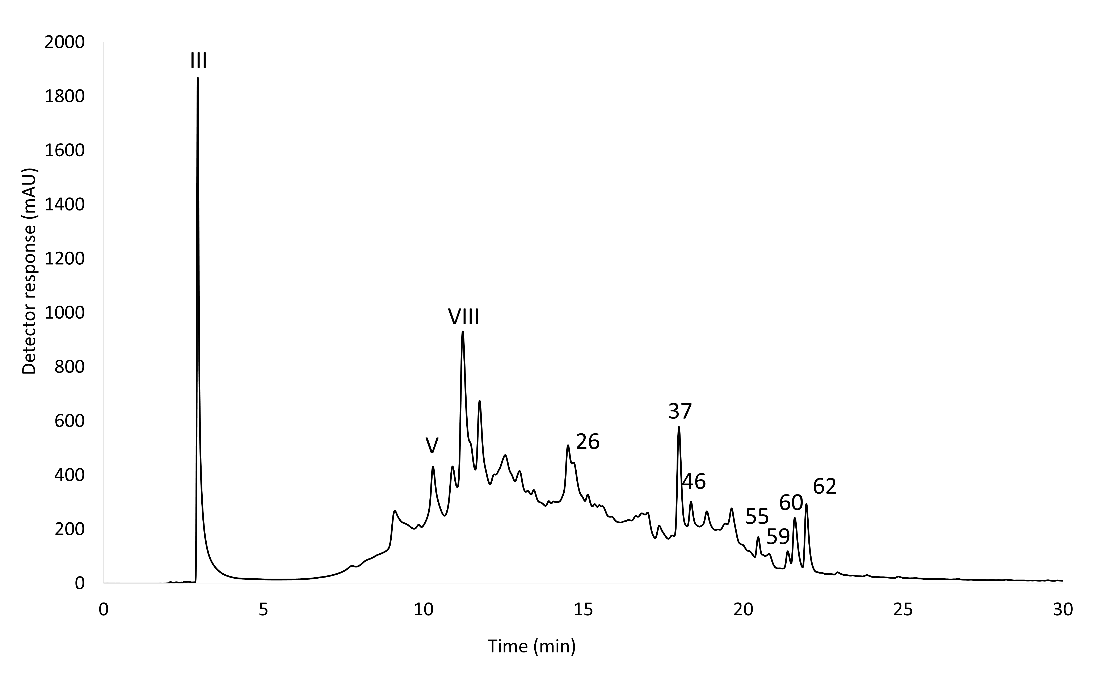


**Figure S5**: HPLC-DAD chromatogram of *F. triphylla* 50% (v/v) ethanolic extract, detection wavelength: 280 nm. Numbering of peaks refers to data shown in Table 1 and 2.


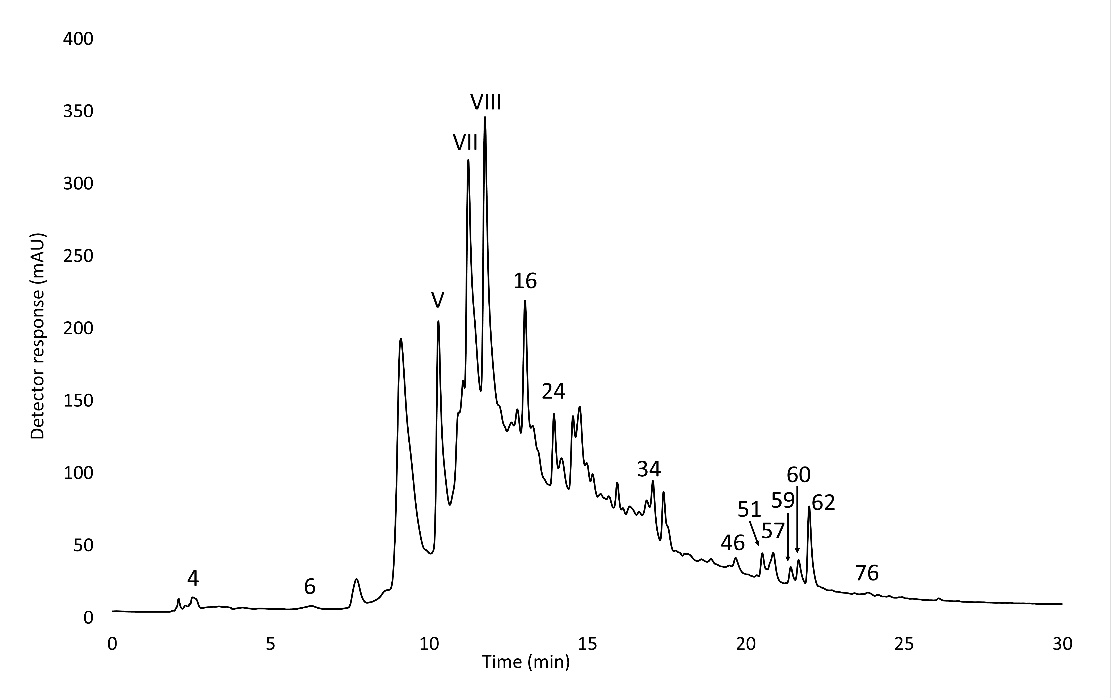


**Figure S6**: HPLC-DAD chromatogram of *F. triphylla* aqueous extract, detection wavelength: 280 nm. Numbering of peaks refers to data shown in Table 1 and 2.


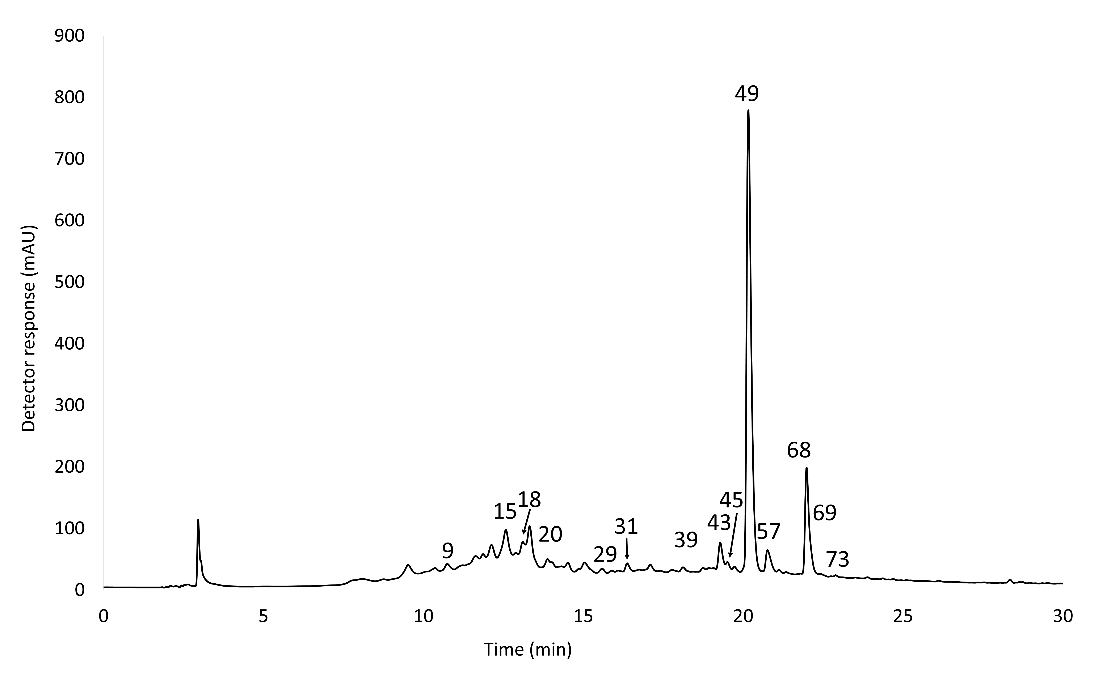


**Figure S7**: HPLC-DAD chromatogram of *L. nummularia* 50% (v/v) ethanolic extract, detection wavelength: 280 nm. Numbering of peaks refers to data shown in Table 1.


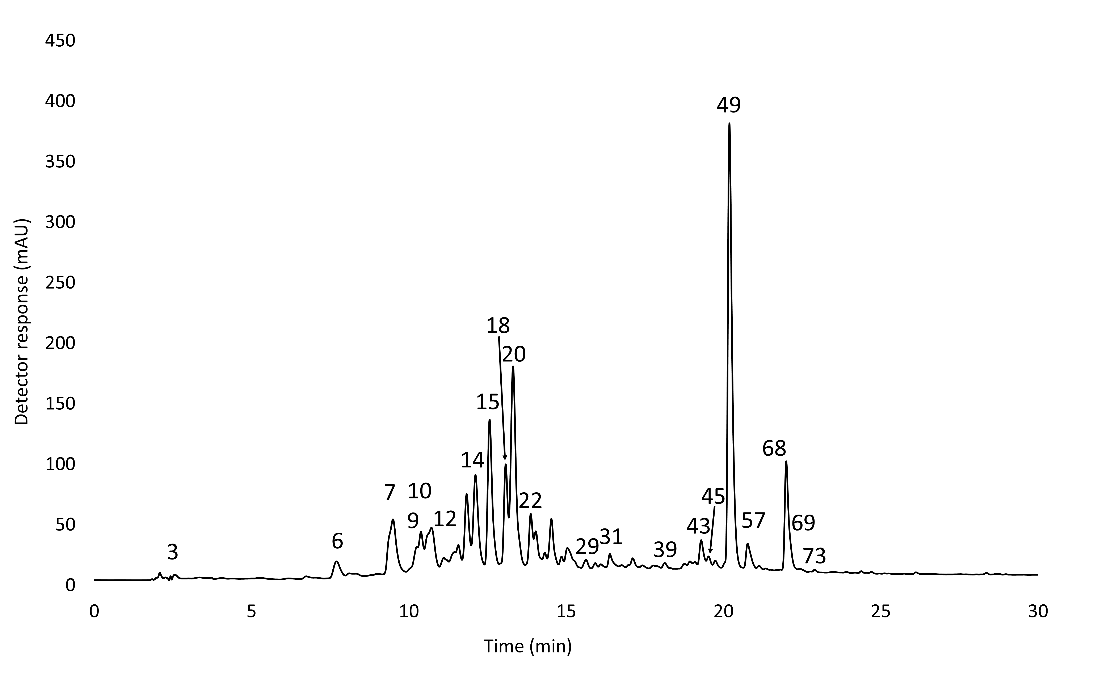


**Figure S8**: HPLC-DAD chromatogram of *L. nummularia* aqueous extract, detection wavelength: 280 nm. Numbering of peaks refers to data shown in Table 1.


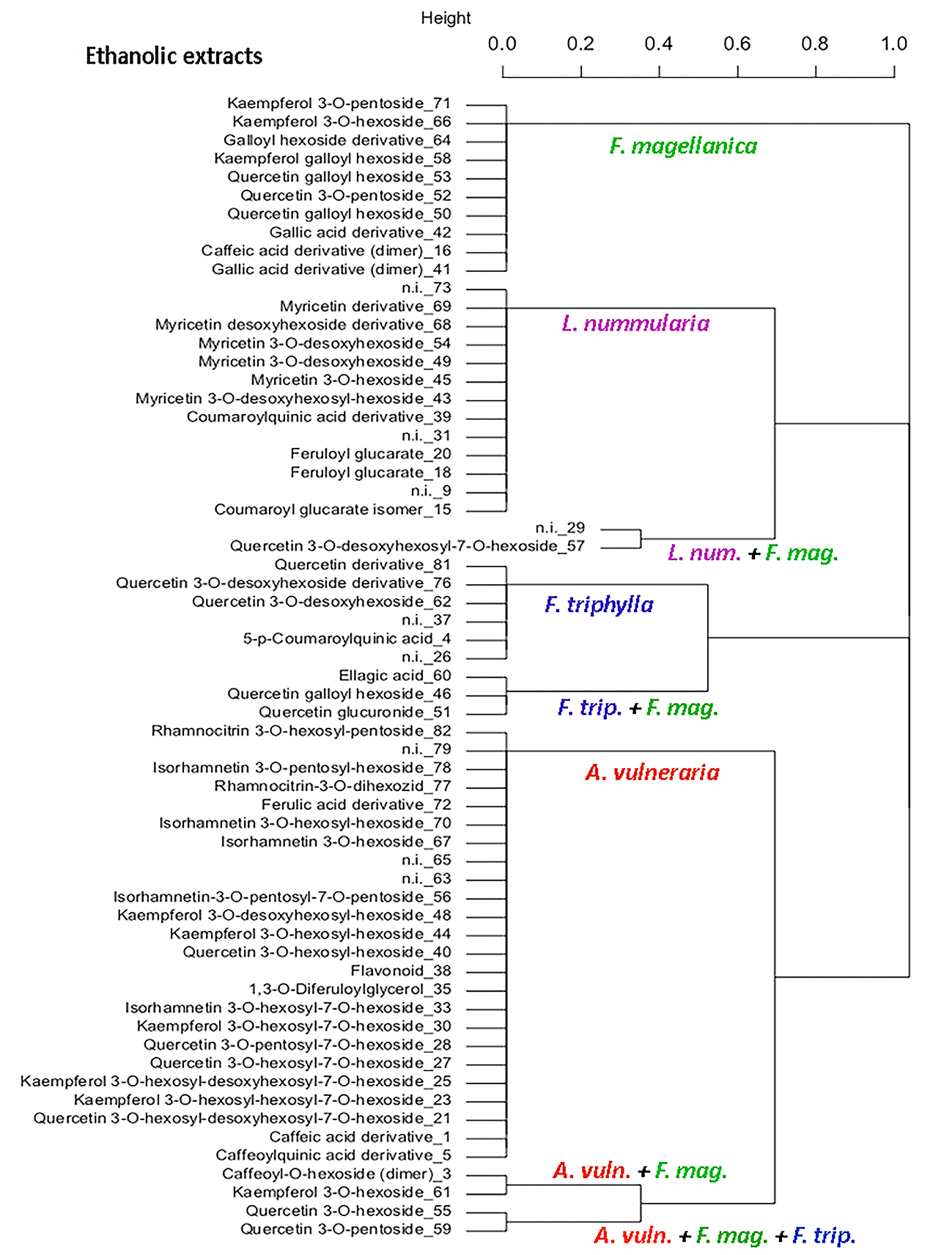


**Figure S9:** Dendrogram of the summarized compound analysis data of 50% (v/v) ethanolic plant extracts.


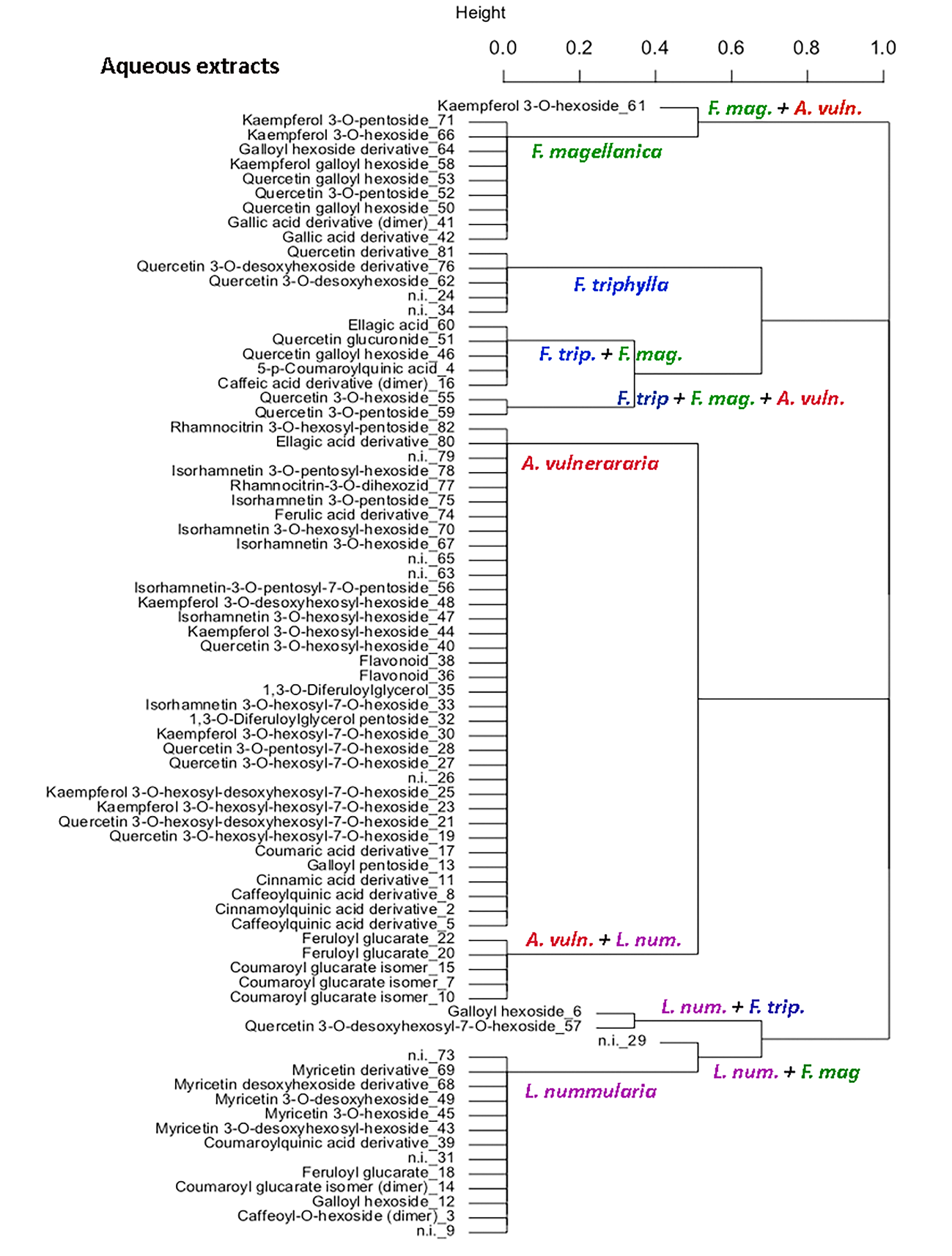


**Figure S10:** Dendrogram of the summarized compound analysis data of aqueous plant extracts.


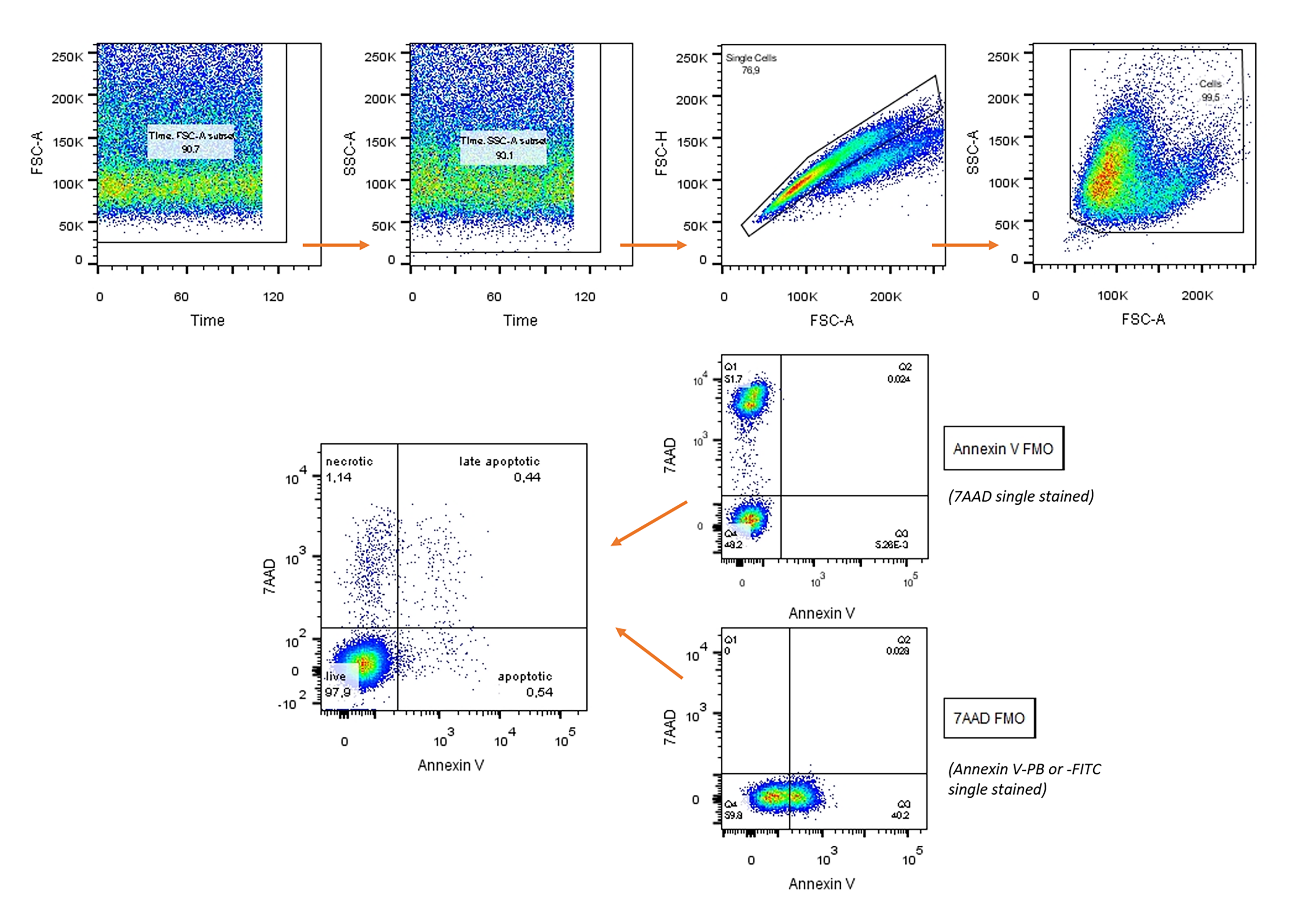


**Figure S11**: Gating strategy of the flow cytometry viability experiments.
