# Supplementary figures and images for: Cytotoxic, Antimicrobial, Antioxidant Properties and Effects on Cell Migration of Phenolic Compounds of Selected Transylvanian Medicinal Plants
Source: Antioxidants (Basel). 2020 Feb 18;9(2):166. doi: 10.3390/antiox9020166 (PMC7070992; doi:10.3390/antiox9020166)

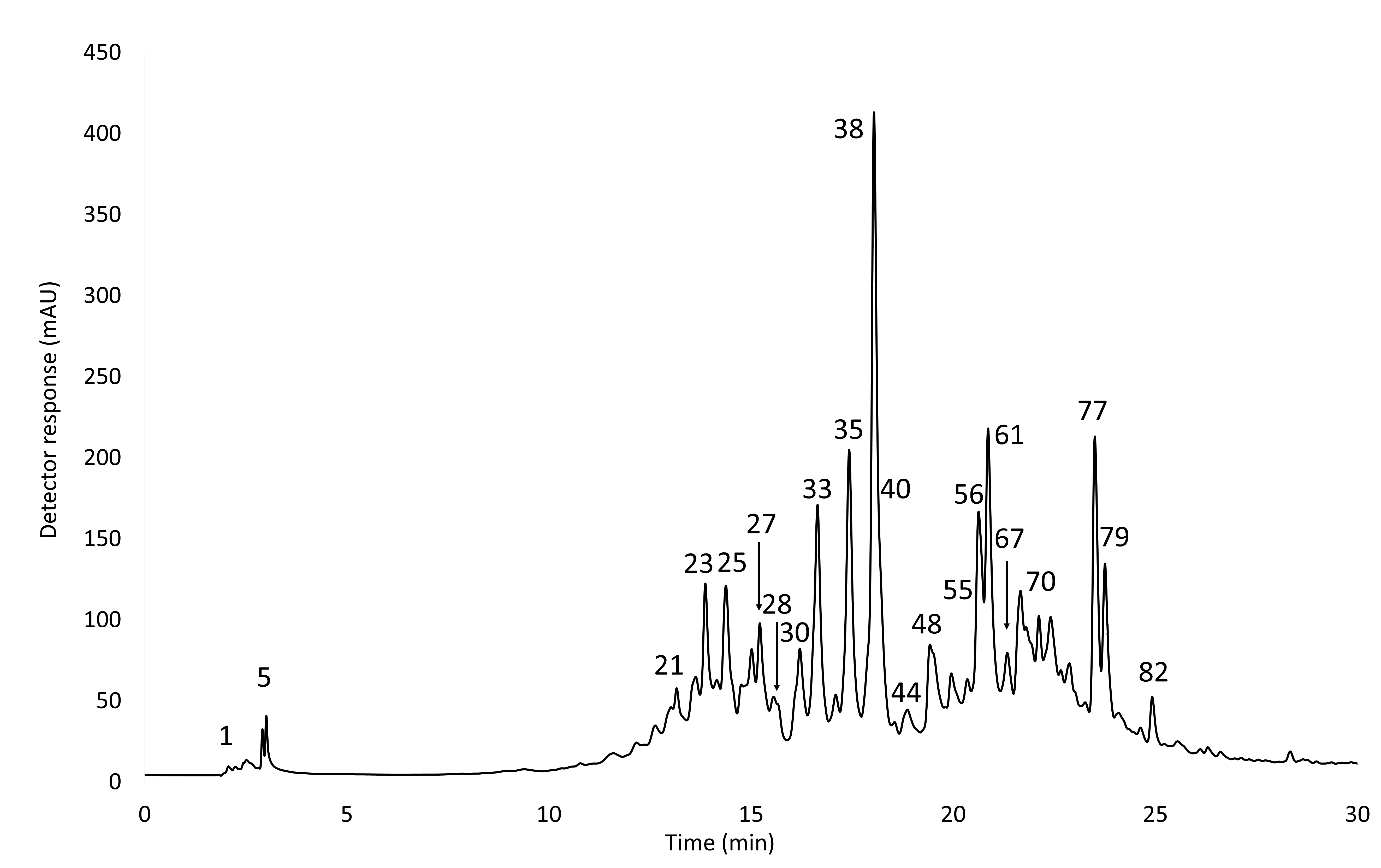

Supplement: Supplementary file 1 [file antioxidants-09-00166-s001.zip › Figure S1.tif]

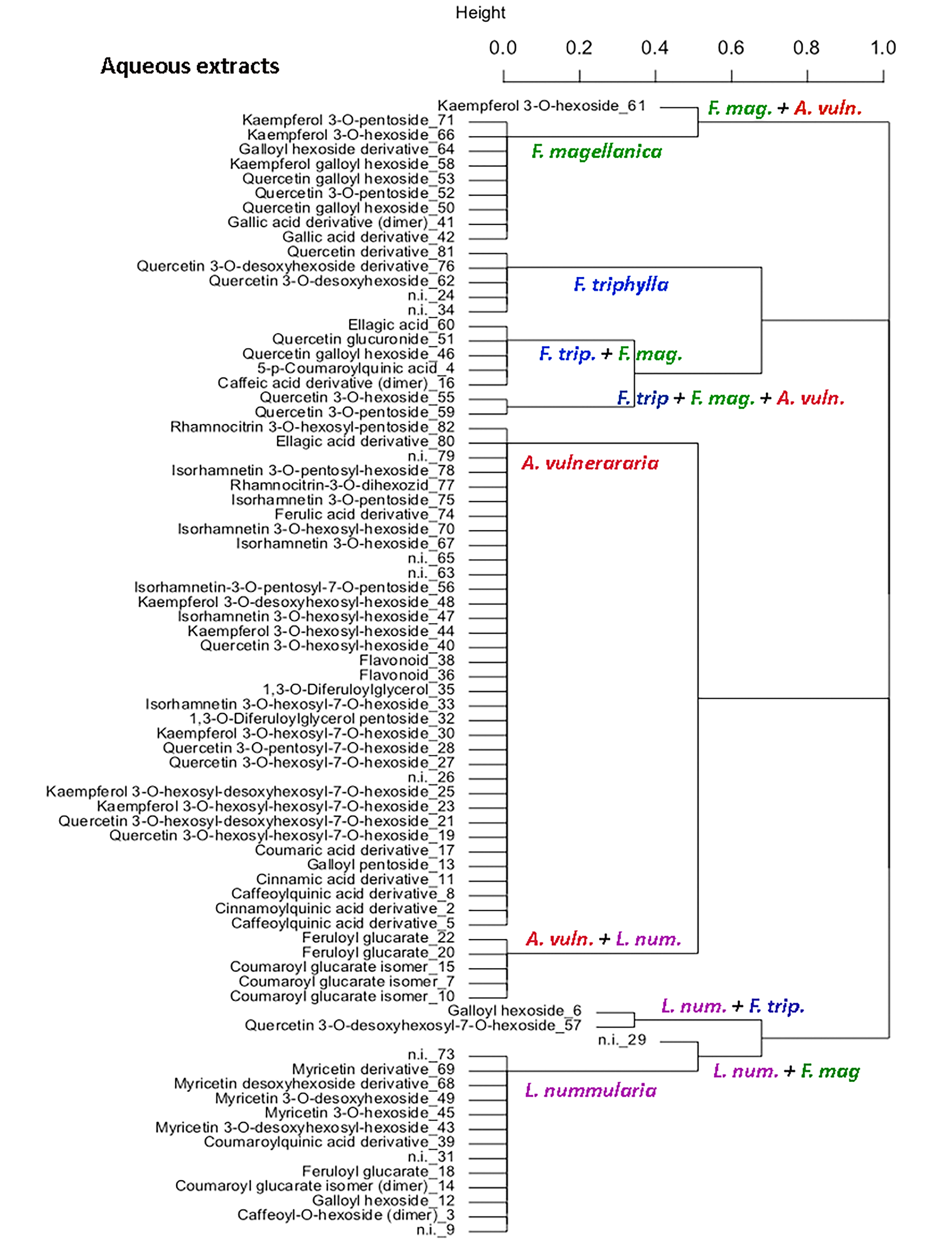

Supplement: Supplementary file 1 [file antioxidants-09-00166-s001.zip › Figure S10.tif]

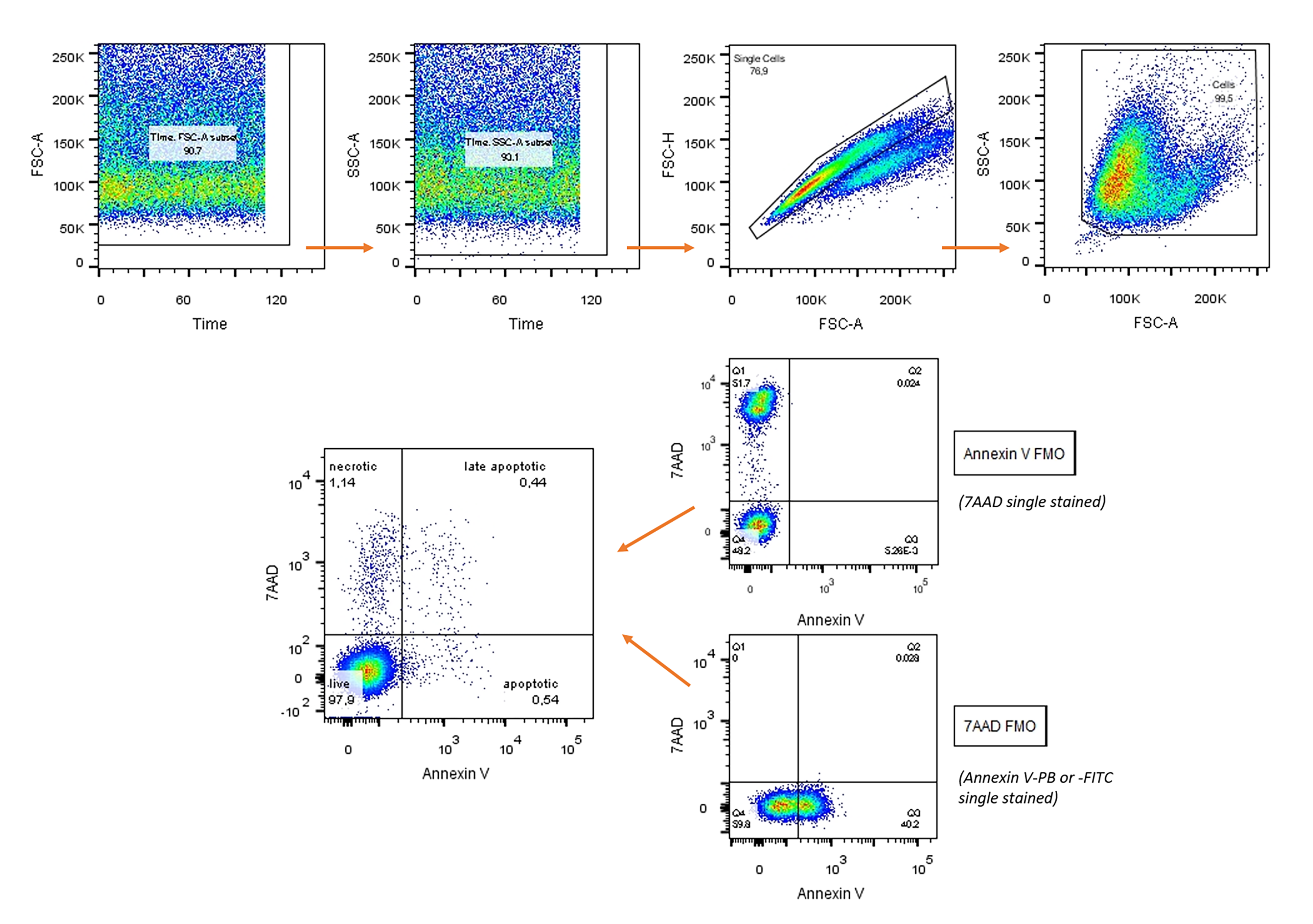

Supplement: Supplementary file 1 [file antioxidants-09-00166-s001.zip › Figure S11.tif]

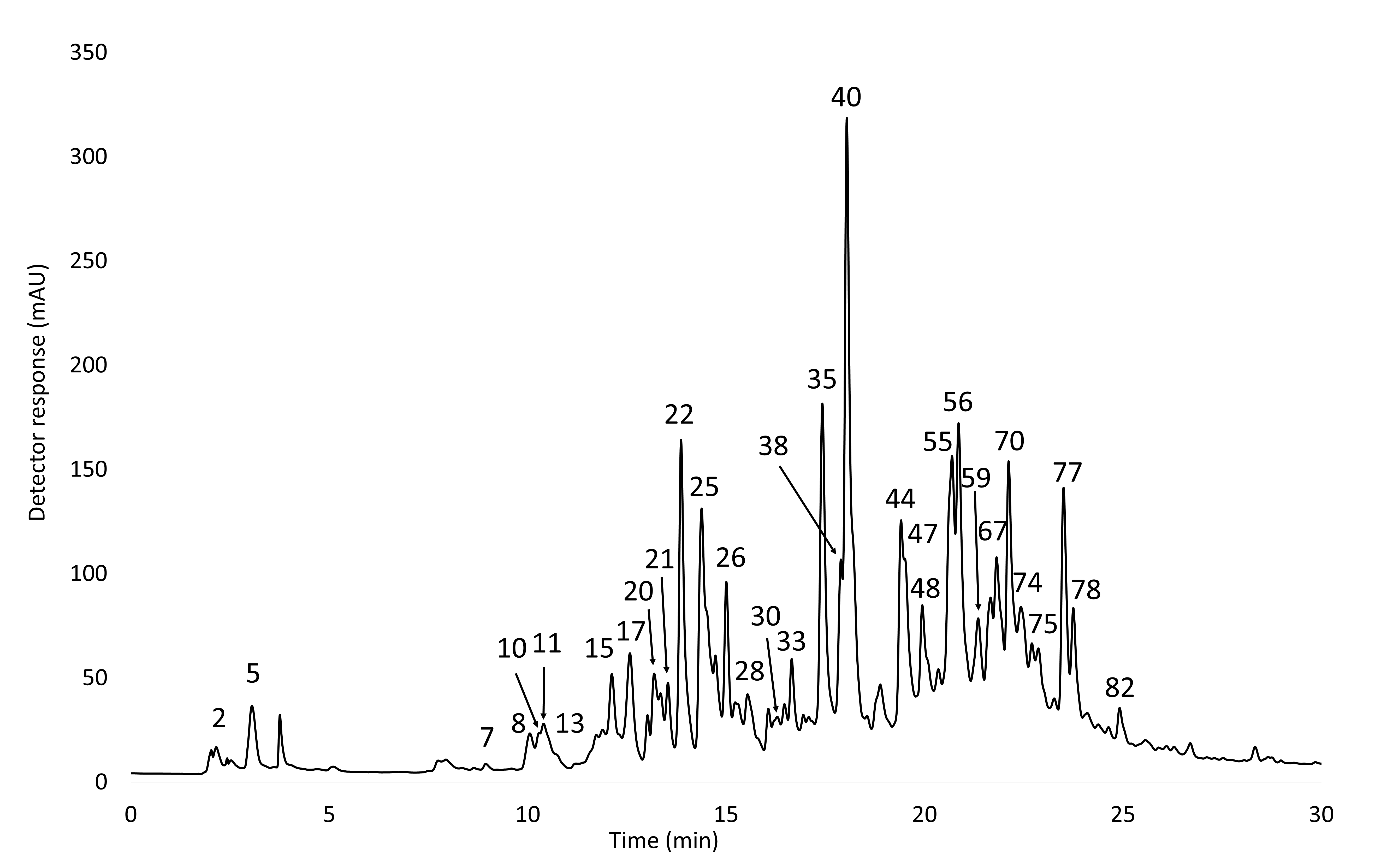

Supplement: Supplementary file 1 [file antioxidants-09-00166-s001.zip › Figure S2.tif]

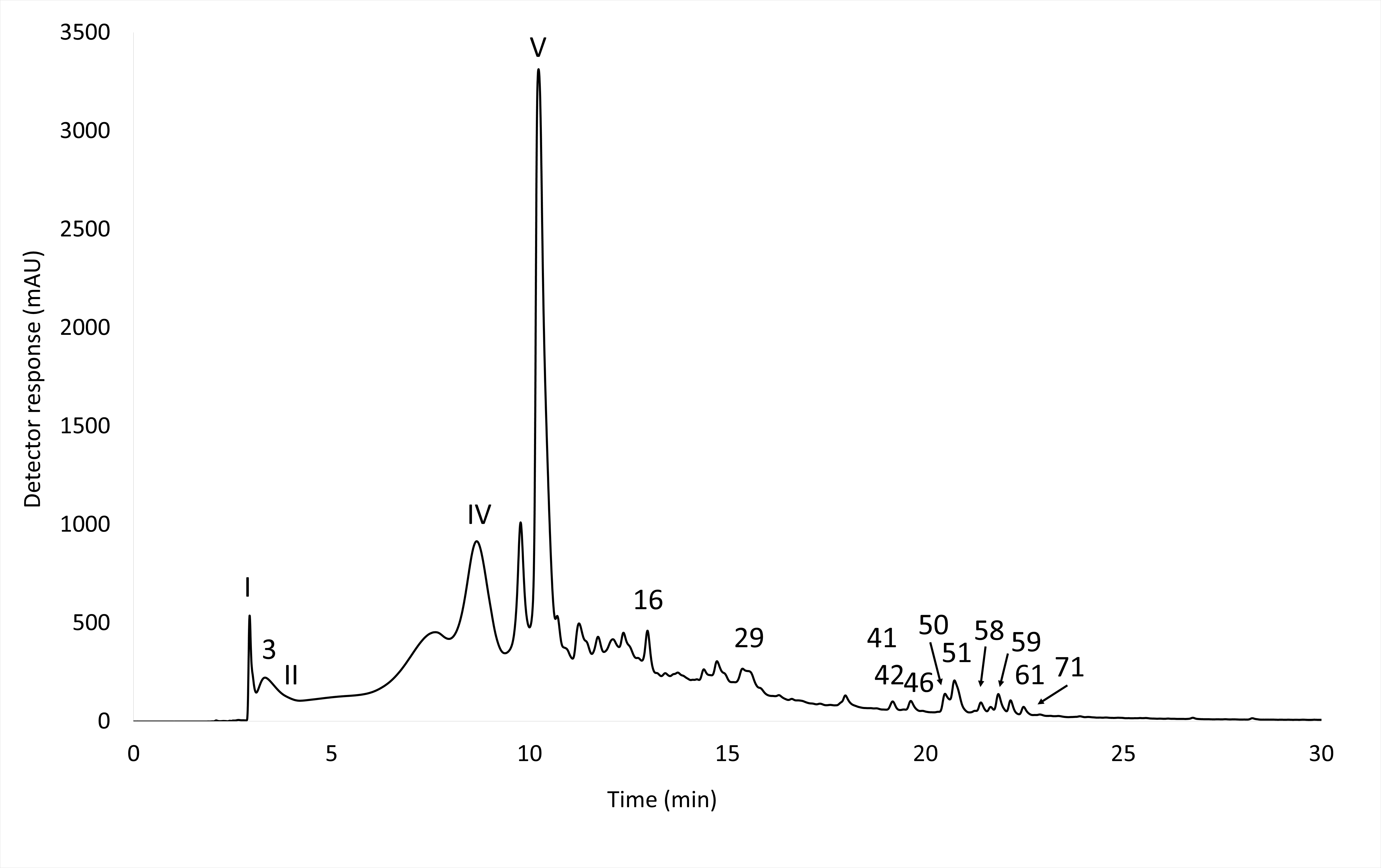

Supplement: Supplementary file 1 [file antioxidants-09-00166-s001.zip › Figure S3.tif]

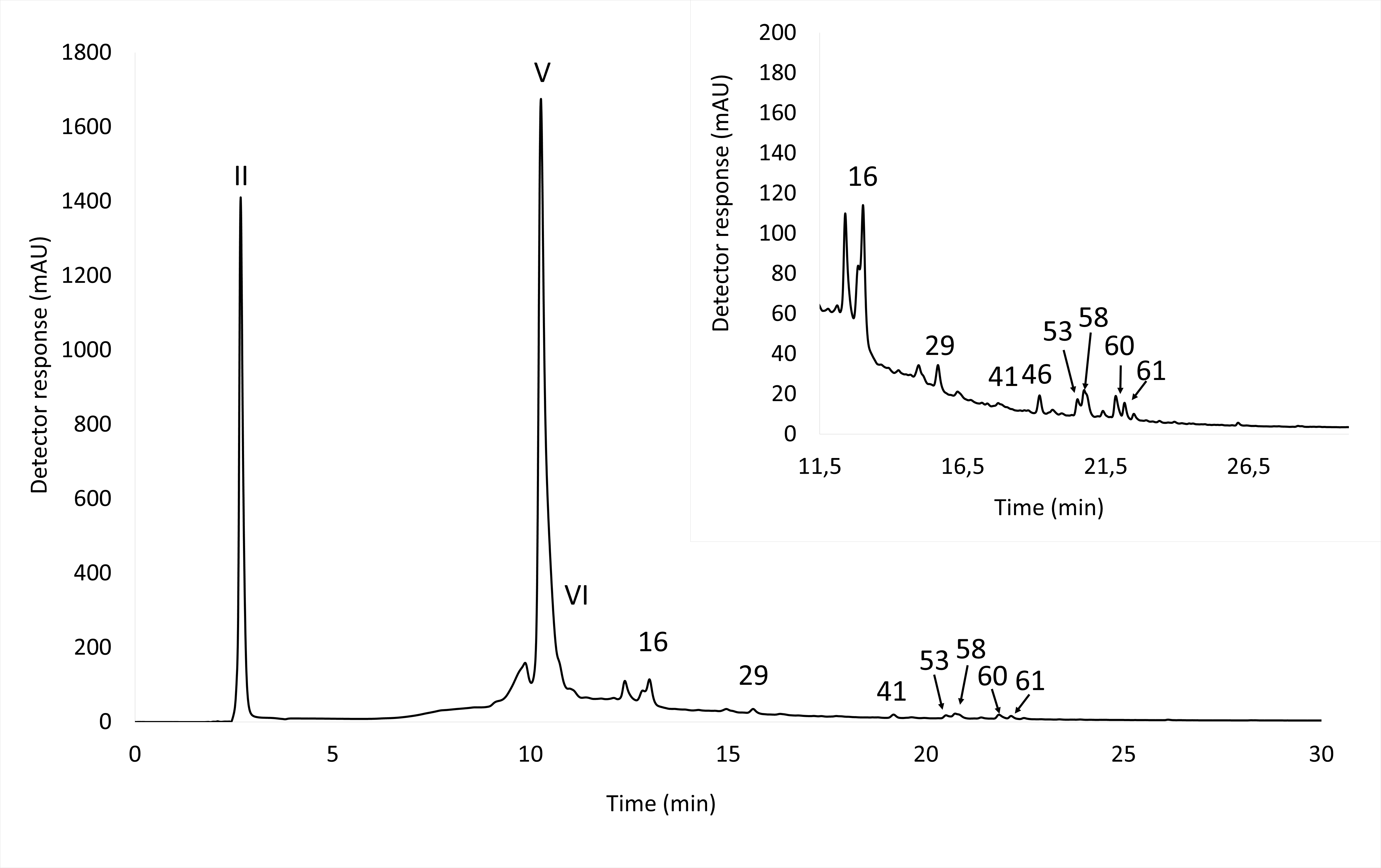

Supplement: Supplementary file 1 [file antioxidants-09-00166-s001.zip › Figure S4.tif]

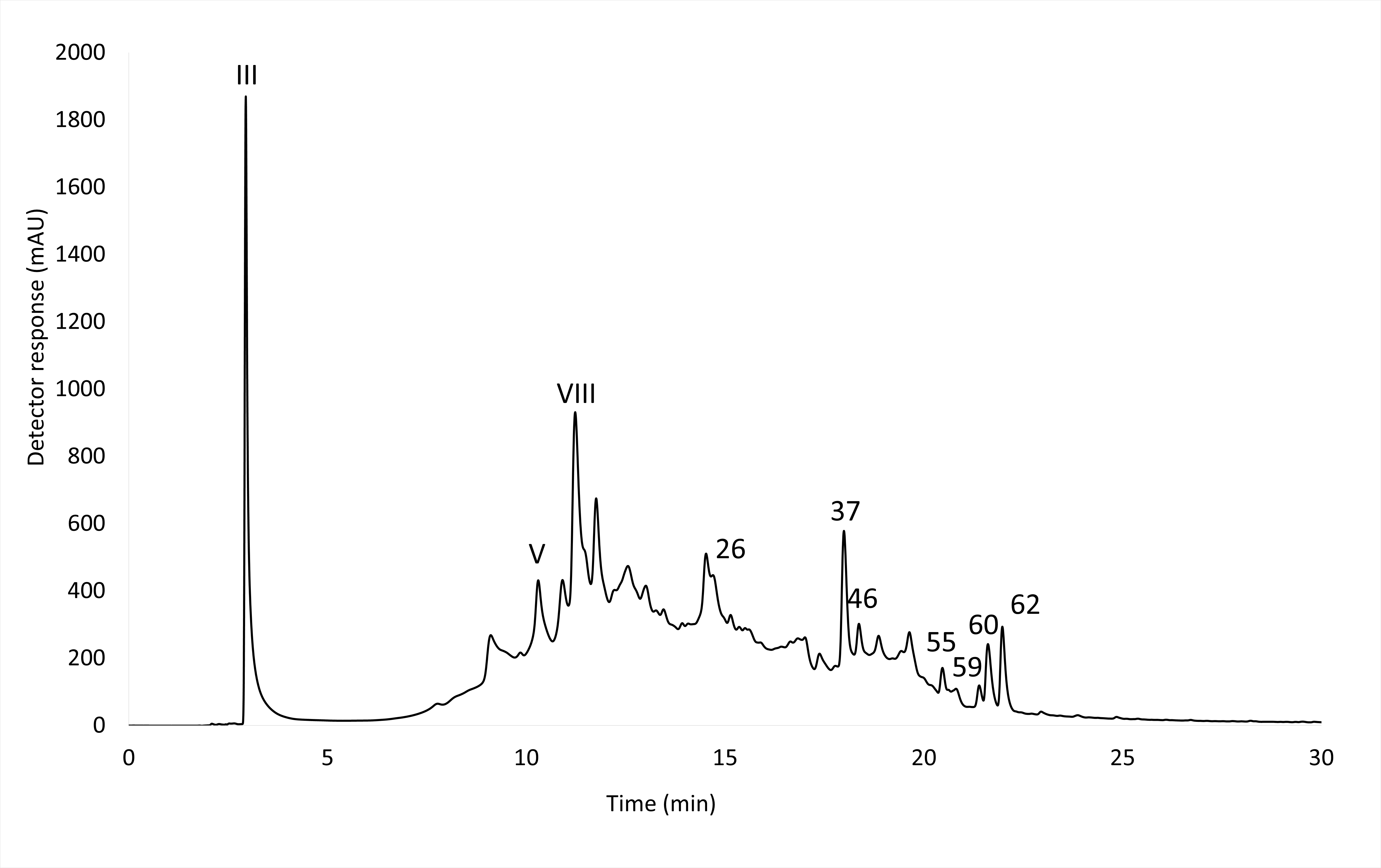

Supplement: Supplementary file 1 [file antioxidants-09-00166-s001.zip › Figure S5.tif]

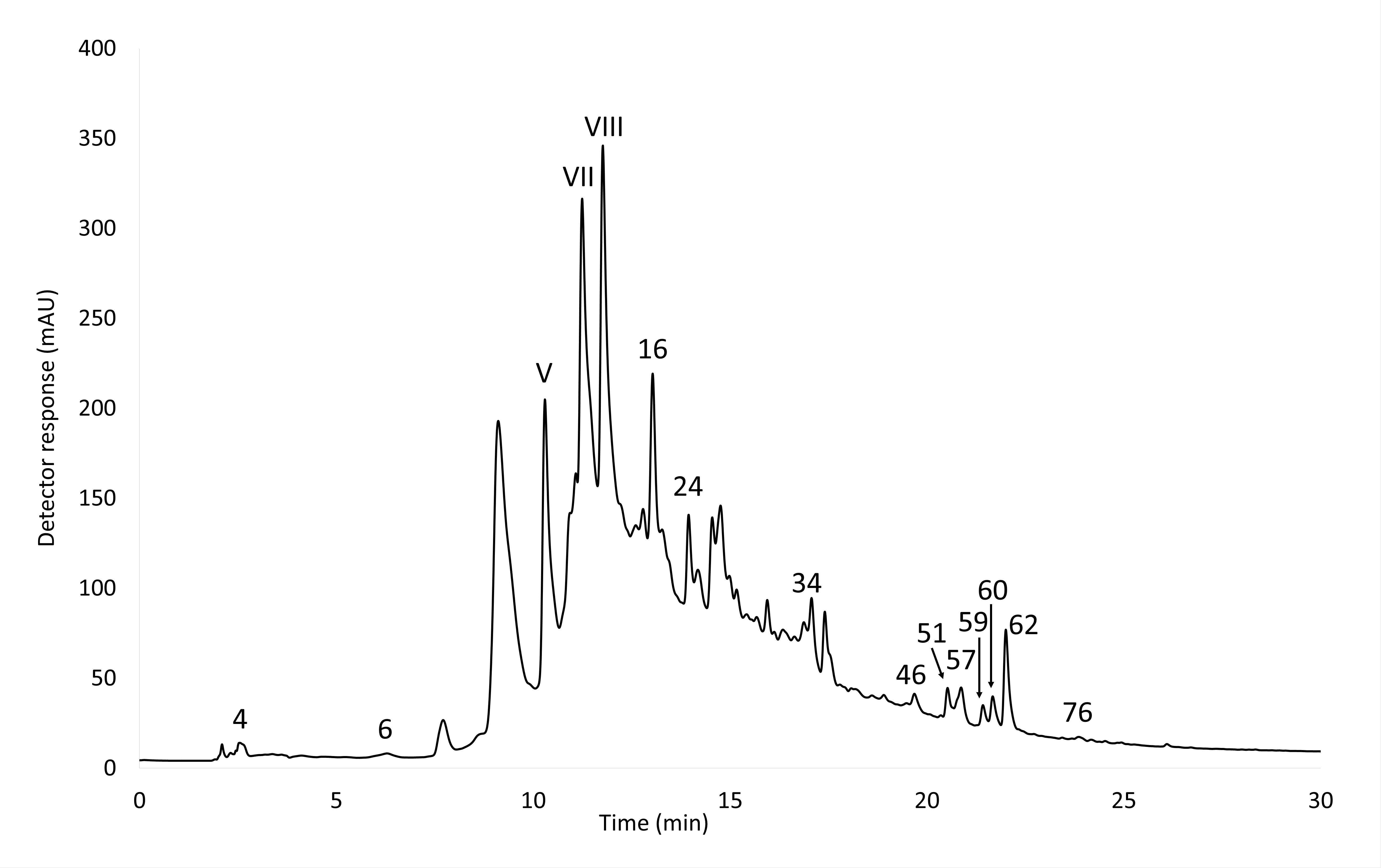

Supplement: Supplementary file 1 [file antioxidants-09-00166-s001.zip › Figure S6.tif]

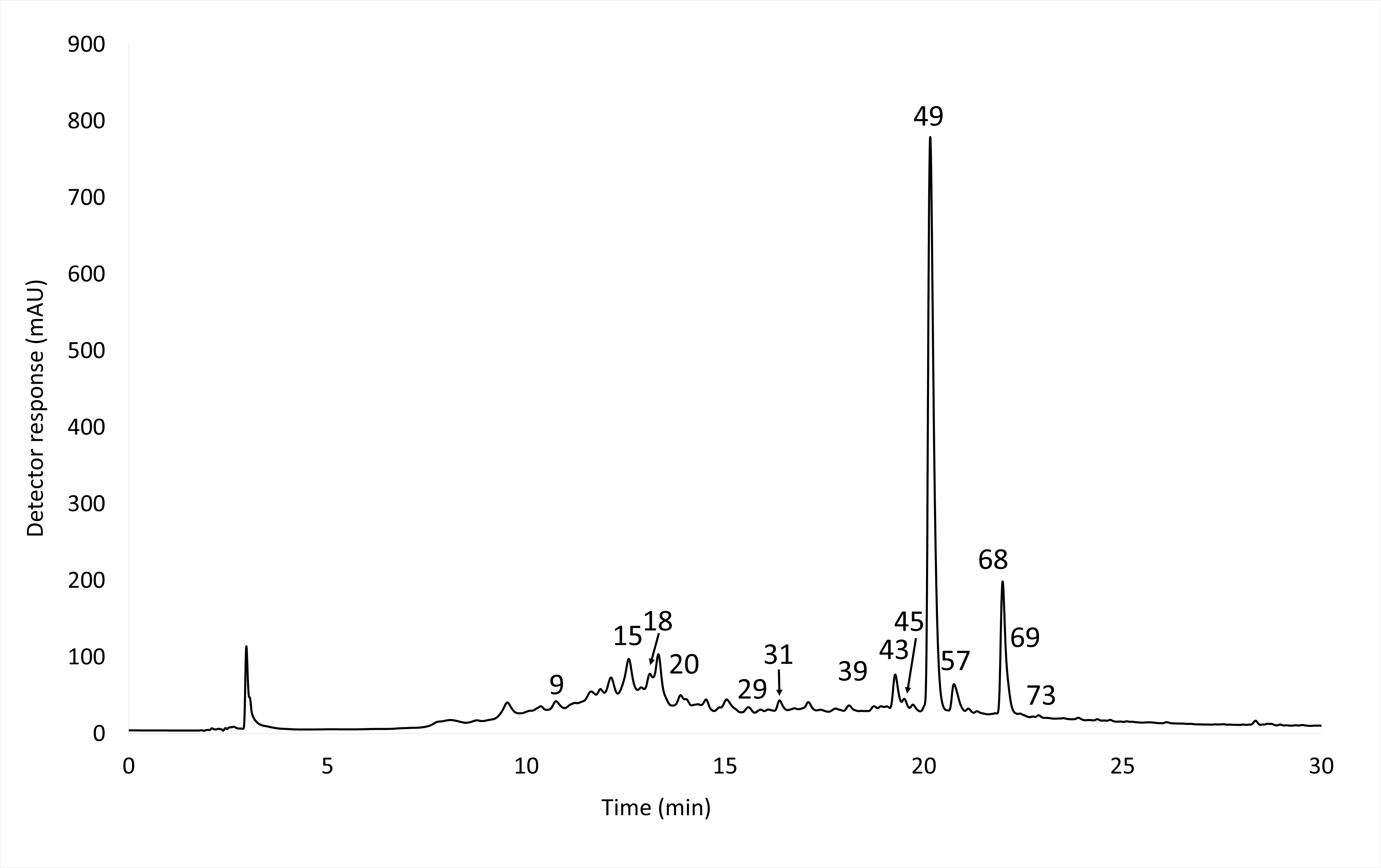

Supplement: Supplementary file 1 [file antioxidants-09-00166-s001.zip › Figure S7.tif]

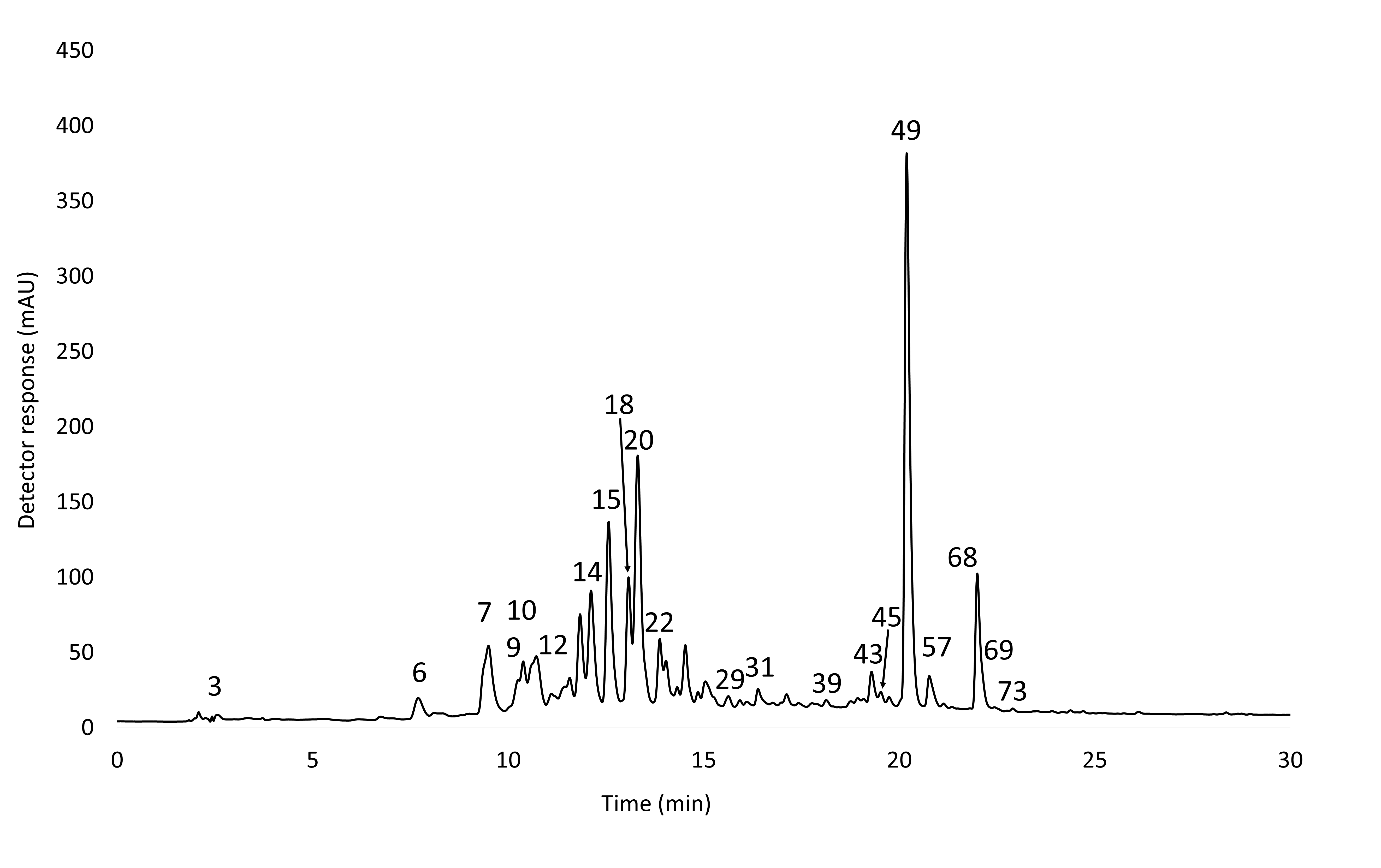

Supplement: Supplementary file 1 [file antioxidants-09-00166-s001.zip › Figure S8.tif]

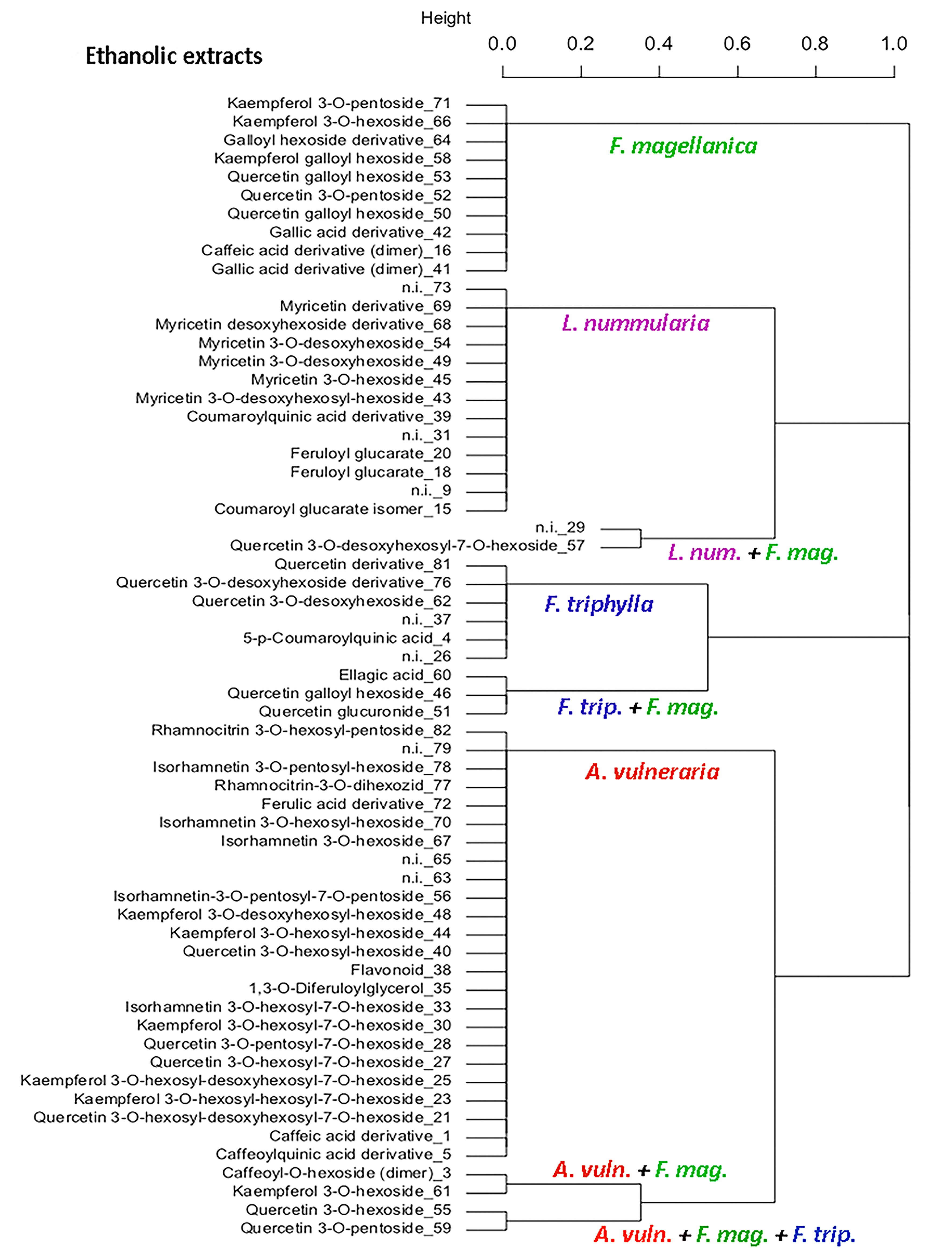

Supplement: Supplementary file 1 [file antioxidants-09-00166-s001.zip › Figure S9.tif]
